# Novel Cyclic Lipopeptides Fusaricidin Analogs for Treating Wound Infections

**DOI:** 10.3389/fmicb.2021.708904

**Published:** 2021-07-23

**Authors:** Joel Gil, Irena Pastar, Richard A. Houghten, Shruti Padhee, Alexander Higa, Michael Solis, Jose Valdez, Cheyanne R. Head, Heather Michaels, Brian Lenhart, Colin Simms, Brandon Williams, Predrag Cudic, Stephen C. Davis

**Affiliations:** ^1^Dr. Phillip Frost Department of Dermatology and Cutaneous Surgery, Miller School of Medicine, University of Miami, Coral Gables, FL, United States; ^2^Torrey Pines Institute for Molecular Studies, San Diego, CA, United States; ^3^Department of Chemistry and Biochemistry Charles E. Schmidt College of Science, Florida Atlantic University, Boca Raton, FL, United States

**Keywords:** *Pseudomonas aeruginosa*, methicillin-resistant *Staphylococcus aureus*, wound healing, porcine (pig) model, wound, biofilm model, cyclic lipopeptides

## Abstract

Both acute and chronic cutaneous wounds are often difficult to treat due to the high-risk for bacterial contamination. Once hospitalized, open wounds are at a high-risk for developing hospital-associated infections caused by multi drug-resistant bacteria such as *Staphylococcus aureus* and *Pseudomonas aeruginosa*. Treating these infections is challenging, not only because of antibiotic resistance, but also due to the production of biofilms. New treatment strategies are needed that will help in both stimulating the wound healing process, as well as preventing and eliminating bacterial wound infections. Fusaricidins are naturally occurring cyclic lipopeptides with antimicrobial properties that have shown to be effective against a variety of fungi and Gram-positive bacteria, with low toxicity. Continuing with our efforts toward the identification of novel cyclic lipopeptides Fusaricidin analogs, herein we report the synthesis and evaluation of the antimicrobial activity for two novel cyclic lipopeptides (CLP), CLP 2605-4 and CLP 2612-8.1 against methicillin resistant *S. aureus* and *P. aeruginosa*, respectively, in *in vivo* porcine full thickness wound model. Both CLPs were able to reduce bacterial counts by approximately 3 log CFU/g by the last assessment day. Peptide 2612-8.1 slightly enhanced the wound healing, however, wounds treated with peptide 2605-4, have shown higher levels of inflammation and impaired wound healing process. This study highlights the importance of identifying new antimicrobials that can combat bacterial infection while not impeding tissue repair.

## Introduction

One of the most significant issues for the field of cutaneous wound care is the prevention and treatment of infections. Persistent wound infections are the most frequent complication of the chronic non-healing wounds (Mustoe, 2004; Sen et al., 2009) and are a leading cause of non-traumatic amputations in diabetic patients (Ivanetich et al., 1976; Boulton et al., 2020). *Staphylococcus aureus*, in both methicillin resistant (MRSA) and sensitive (MSSA) form, and *Pseudomonas aeruginosa* are the most common culprits of infections in both acute and chronic wounds (Aronson et al., 2006; Murray, 2008; Murray et al., 2008; Wolcott et al., 2016; Tomic-Canic et al., 2020). Furthermore, these pathogens are common causes of hospital-acquired infections caused by multi-drug resistant (MDR) strains. Discovering adequate and effective treatments for infections caused by these bacteria is challenging, not only due to bacterial resistance to antimicrobials, but also because of the ability for these bacteria to form biofilms that provide further protection from host immune responses and antimicrobial treatment (Donlan, 2001; Lipsitch, 2001). Currently there are few combination antimicrobial modalities such as daptomycin with moxifloxacin plus clarithromycin that have shown the potential capacity against *S. aureus*, such as MSSA (SH1000) and MRSA (N315), to prevent and disrupt the formation of biofilms (Raad et al., 2007; Parra-Ruiz et al., 2010). This prompted the search for antimicrobials that utilize different modes of action compared to conventional antibiotics and may also belong to a new class of drugs, thereby reducing the progression of antibiotic resistance.

The relatively low number of novel antimicrobials meeting FDA approval has extended the search outside of typical antimicrobial peptides (Boucher et al., 2013). Naturally occurring compounds known as cyclic lipopeptides (CLP) have become a source for new antimicrobial agents due to their structural diversity and high potency (Giuliani et al., 2007; Roongsawang et al., 2011; Bionda et al., 2013b). The CLP daptomycin (Cubicin^®^, Cubist Pharmaceuticals Inc., MA, United States) has been approved in the United States, European Union, and Canada as an effective treatment for complicated skin and soft tissue infections that are caused by Gram-positive bacteria, such as MDR *S. aureus*, *Streptococcus pyogenes*, *Streptococcus agalactiae*, and *Enterococcus faecalis* (Lipsitch, 2001; Bionda and Cudic, 2011). The application of this drug has been limited due to emerging bacterial strains with reduced susceptibility and ineffectiveness when used to treat biofilms and chronic infections (Woodford, 2003; Kern, 2006).

Fusaricidins are positively charged CLP isolated from *Paenibacillus polymyxa*, and are structurally distinct from usual cationic antimicrobial peptides (CAMPs) (Kurusu et al., 1987; Kajimura and Kaneda, 1996, 1997; Kuroda et al., 2000; Raad et al., 2007). Typical CAMPs use positive charges from amino acids, such as arginine, lysine and histidine, to target the bacterial membrane (Hancock and Lehrer, 1998; Wu and Li, 2005; Peschel and Sahl, 2006). Fusaricidins differ from most CAMPs as there is only a single positive charge at the termini of their lipidic tails (Giuliani et al., 2007; Bionda and Cudic, 2011; Bionda et al., 2013b). Thus, fusaricidins have been shown to use an alternate mode of action against microbes, possibly providing a novel class of antimicrobial peptides (Bionda et al., 2013a). Mice injected with fusaricidins show low acute toxicity, and further structural modifications of these compounds could potentially lead to potent analogs with greater stability and further reduced non-specific toxicity (Bionda et al., 2012, 2013b).

Due to the expedient synthesis of these CLPs using the tea bag approach previously reported (Bionda et al., 2016), fusaricidin derivatives with several unnatural amino acids were synthesized and tested *in vitro*. These modifications have been shown to inhibit development of resistance, as peptides incorporating unnatural amino acids are less sensitive to proteases and therefore have longer half-lives (Blaskovich, 2016).

To identify the efficacy of novel compounds to treat cutaneous wounds and associated infections, this study examined the antimicrobial effects of two CLPs as well as their influence on wound healing. The key to the development of potent non–toxic antibacterial agents lies in the ability to balance hydrophobicity and positive charge. Hence, in pursuit of better drug candidates we introduced hydrophobic, unnatural amino acid residues or added more positively charged residues to the CLPs. Based on *in vitro* results, peptide 2605-4 was used to study these effects against methicillin-resistant *S. aureus* (MRSA) and peptide 2612-8.1 was used to study *P. aeruginosa* infection *in vivo* (Mertz et al., 1999; Davis and Mertz, 2008; Nusbaum et al., 2012). Pigs were used as the model animal due to the morphological similarities between porcine and human skin and the wound healing process closely resembling human tissue repair (Meyer et al., 1978; Sullivan et al., 2001; Perez and Davis, 2008). Wound healing parameters such as re-epithelialization, epithelial thickness, white cell infiltrates and granulation tissue formation were examined, as well as the expression of several pro-inflammatory cytokines. The ability of these peptides to reduce bacterial infection while promoting wound healing was indicated by microbiological wound assessment, gene expression of pro-inflammatory cytokines, and the re-epithelialization of wounded tissue. While CLP 2612-8.1 was effective in reducing *P. aeruginosa* load in *in vivo* wound model, CLP 2605-4 has reduced wound infection caused by MRSA but impeded wound healing process. Our study highlights the importance of identifying novel antimicrobials that can combat wound infections while not interfering with tissue repair process.

## Materials and Methods

### Peptide Synthesis

**FIGURE 1:**
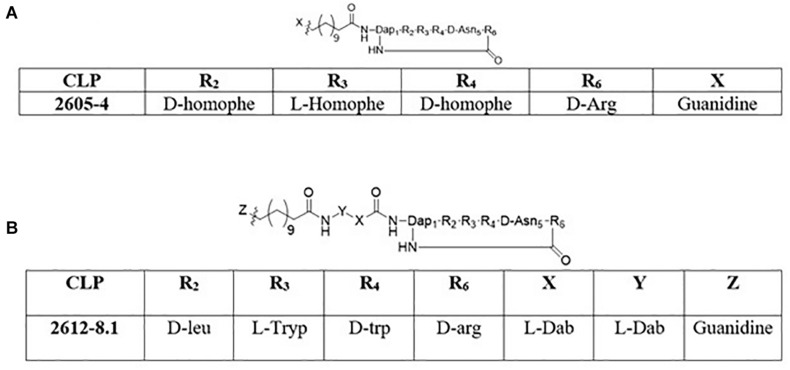
Structures of cyclic lipopeptides: **(A)** 2605-4 **(B)** 2612-8.1.

The synthesis of cyclic peptides 2605-4 and 2612-8.1 was performed following the strategy described by Bionda and Cudic (2011). The 2605-4 peptide (Figure 1A) macrocycle is composed of Fmoc-D-aspartic acid α-allyl ester, Fmoc-D-homophenylalanine, Fmoc-L-homophenylalanine, Fmoc-D-homophenylalanine, *N*^α^ –Fmoc-*N*^β^ -4-methyltrityl-L-2,3-diaminopropionic acid and *N*^α^ -Fmoc-*N*^ω^ -(2,2,4,6,7-pentamethyldihydrobenzofuran-5-sulfonyl)-D-arginine (Chemimpex), respectively. The Fmoc-D-aspartic acid α-allyl ester, Fmoc-D-homophenylalanine, Fmoc-L-homophenylalanine and Fmoc-D-homophenylalanine, *N*^α^ –Fmoc-*N*^β^ -4-methyltrityl -L-2,3-diaminopropionic acid were added sequentially onto the solid support Tentagel XV RAM resin (RAPP Polymere) followed by addition of the Fmoc-12-aminododecanoic acid (Chemimpex) using standard Fmoc chemistry. The Fmoc-12-amino dodecanoic acid tail was then coupled with 1,3-Di-Boc-2-(trifluoromethylsulfonyl)guanidine with 4 equivalents of triethylamine (TEA) in dichloromethane (DCM) to cap the lipid tail. Following the addition of the guanidine cap, the 4-methyltrityl (Mtt) protecting group from the diaminopropionic acid residue was cleaved using 1% trifluoroacetic acid (TFA)/3% triisopropylsilane (TIS)/96% DCM and the last residue (*N*^α^ -Fmoc-*N*^ω^ -(2,2,4,6,7-pentamethyldihydrobenzofuran-5-sulfonyl)-D-arg ini ne) is coupled. Once the last residue was coupled onto the solid support, the allyl ester protecting group was cleaved by using 3 equivalents of palladium-tetrakis(triphenylphosphine) in a solution of DCM: acetic acid: *N*-methylmorpholine (20:2:1) volumetric ratio. Post removal of the allyl protecting group, the Fmoc protecting group from the D-arginine residue was removed using a solution of 20% piperidine/DMF for 10 min (repeated twice) and the cyclization of the lipopeptide was completed on solid support using (benzotriazol-1-yloxy) tripyrrolidinophosphonium hexafluorophosphate (PyBop), 1-hydroxybenzotriazole (HOBt) and *N*,*N*-diisopropylethylamine (DIEA) in dimethylformamide (DMF). Following overnight cyclization, the bags were rinsed with anhydrous DMF and DCM and dried under vacuum. The final cyclic lipopeptide (CLP) was cleaved from the resin using 88% TFA/6% water/6% TIS. The cleaved oligomers were then transferred to scintillation vials, frozen and lyophilized. Compounds were then reconstituted in 50% acetonitrile and water, frozen and lyophilized three more times.

The 2612-8.1 macrocycle is composed of Fmoc-D-aspartic acid α-allyl ester, Fmoc-D-tryptophan, Fmoc-L-tryptophan, Fmoc-L-leucine, *N*^α^ –Fmoc-*N*^β^ -4-methyltrityl-L-2,3-diaminopropionic acid and *N*^α^ -Fmoc-*N*^ω^ -(2,2,4,6,7-pentamethyl-dihydrobenzofuran-5-sulfonyl)-D-argin ine (Chemimpex), respectively. The synthesis of 2612-8.1 (Figure 1B) involved sequential insertion of two additional exocyclic residues comprising of *N*^α^ -Fmoc-*N*^γ^ -Boc-L-2,4-diaminobutyric acid (linker), between the macrocycle and the Fmoc-12-amino dodecanoic acid using standard Fmoc chemistry. Post addition of the Fmoc-12-amino dodecanoic acid similar methodology (mentioned above) was used to complete the capping of the C-12 tail with guanidine functional group, followed by Mtt removal, addition of *N*^α^ -Fmoc-*N*^ω^ -(2,2,4,6,7-pentamethyl-dihydrobenzofuran-5-sulfonyl)-D-arginine, allyl deprotection, Fmoc deprotection and lastly cyclization. The CLP was then cleaved from the solid support following identical strategy as above and subjected to 3 rounds of lyophilization. Purity and identity of the peptides were also verified by HPLC, LC-MS and MALDI-TOF (Supplementary Data).

### *In vitro* Antibacterial Activity

The antibacterial activities of the new unpublished series of CLPs were initially examined against one Gram-positive and two Gram-negative bacterial strains: *S. aureus* Mu50 (MRSA) ATCC 700699 (Song et al., 2020), *P. aeruginosa* ATCC-27853 (Dean et al., 2011) and *A. baumannii* ATCC 19606 (Krasauskas et al., 2019; Selvaraj et al., 2020), respectively. Antibacterial assays were performed according to the microbroth dilution method as outlined by the Clinical and Laboratory Standards Institute (Wiegand et al., 2008). To perform the broth microdilution assay a single colony was grown in 5 mL of Tryptase Soy Broth for about 4 h (Dean et al., 2011; Krasauskas et al., 2019; Selvaraj et al., 2020; Song et al., 2020). The bacterial suspension was then diluted using fresh media to 10^5^–10^6^ Colony Forming Units (CFU)/mL. This was followed by serial dilution of cyclic peptides and the control antibiotics included in the study for the purpose of preparing different concentrations of each antimicrobial component. Vancomycin and Colistin were tested alongside CLPs and served as control antibiotics. The diluted peptides as well as control antibiotics used in this assay ranged from 3.1 to 100 μg/mL. Plates were then loaded with 10 μL aliquots of serial dilutions of the cyclic peptides/antibiotics and 90 μL diluted bacterial suspension. Plates were then incubated at 37°C overnight and after 18 h of incubation, minimum inhibitory concentration that resulted in 90% population reduction (MIC 90) were recorded. All the tests were performed in technical duplicates and were repeated as three independent experiments.

### Experimental Animals

The following study was reviewed and approved by the University of Miami’s Institutional Animal Care and Use Committee and the US Army’s Animal Care and Use Committee. The study was performed according to the University of Miami’s Department of Dermatology and Cutaneous Surgery’s Standard Operating Procedures. Swine were used as the research animal due to the morphological similarities between porcine and human skin (Meyer et al., 1978; Sullivan et al., 2001; Perez and Davis, 2008). Six female *Sus Scrofa* Yorkshire specific pathogen-free *animals* (SPF; Looper Farms, North Carolina) weighing 35–45 kg were housed for 2 weeks preceding the experiment, to allow for acclimation to the environment. Two pigs were used to evaluate the antimicrobial effects of CLP on MRSA wound infections, two pigs were used to evaluate the effects against *P. aeruginosa*, and two additional animals were used to study the effects of CLP on the wound healing process. Animals were fed a non-antibiotic feed *ad libitum* and housed individually in our animal care facilities (American Association for the Accreditation of Laboratory Animals accredited) with a controlled temperature (19–21°C) and light schedule (12 h/12 h LD).

### Animal Preparation and Wounding

Animals were anesthetized during all procedures with telazol (1.4 mg/kg), xylazine (2.0 mg/kg) and atropine (0.05 mg/kg) intramuscularly (IM) and were given a combination of inhaled isoflurane/oxygen. Proceeding anesthesia, hair on the backs and flanks of the animals were trimmed with standard animal clippers. The shaved skin on both sides of the animal was washed with a non-antibiotic soap (Neutrogena^®^) and sterile water. For the evaluation of antimicrobial effects, thirty-six full thickness wounds were created on the paravertebral and thoracic area of each animal with an 8 mm punch biopsy. Eight wounds were randomly assigned to four treatment groups. For the wound healing experiment, forty-eight full thickness wounds were created on each animal (*n* = 2) using an 8 mm punch biopsy. Twelve wounds were randomly assigned to each treatment groups.

### Wound Inoculation

Fresh cultures of MRSA USA-300 and *P. aeruginosa* 09-010 (military combat isolate acquired as a gift from Dr. Lee Cancio) were used for this study separately, but both found in polymicrobial wound infections (Pastar et al., 2013). Serial dilutions were made until a concentration of 10^6^ CFU/ml was achieved, as determined by optical density measurements and by platting onto selective media to quantify the exact number of viable organisms used for wound infections. The inoculum was then vortexed and each wound was inoculated with a 25 μl aliquot of the inoculum suspension, deposited into a glass cylinder (22 mm in diameter) in the center of each wound. The aliquoted suspension was then lightly scrubbed into the wound site for 10 s using a sterile Teflon spatula. Each wound was individually covered with a polyurethane film dressing (Tegaderm, 3M, Saint Paul, MN, United States) for 24 h to allow biofilm formation (Davis et al., 2008).

### Treatment Regimen

Microbiology study: after 24 h the polyurethane film dressings were removed and wounds were randomly assigned to various treatment groups. Both peptides were prepared in dimethyl sulfoxide (DMSO) at 20 mg/ml, which was the optimal concentration that produced the best antimicrobial effects for *in vivo* pilot studies (data not shown). For MRSA infected wounds, the treatments groups were: (A) DMSO + Peptide 2605-4; (B) DMSO (vehicle control); (C) Mupirocin (positive control, Taro Pharmaceutical Industries, Ltd.); and (D) untreated control. For *P. aeruginosa* inoculated wounds, the treatment groups were: (A) DMSO + Peptide 2612-8.1; (B) DMSO (vehicle control); (C) Silver sulfadiazine (positive control; Ascend Laboratories, LLCS); and (D) untreated control. All treatment groups including untreated were covered with a polyurethane film dressing to avoid cross contamination. Treatments were secured with surgical tape and the entire animal was loosely wrapped with self-adhering bandages (Coban; 3M). Wounds were treated daily with 100 μL peptide solutions by pipette to cover the wound and surrounding unwounded skin. Silver sulfadiazine (SSD) and Mupirocin positive control wounds were treated with 100 μL using a syringe. Dressings were replaced after each treatment application.

For the evaluation of the effects on wound healing 36 wounds were randomly assigned to one of the following treatment groups on each animal: (A) Peptide 2605-4 in DMSO [20 mg/mL]; (B) Peptide 2612-8.1 in DMSO [20 mg/mL]; (C) DMSO (vehicle control); and (D) untreated control. Treatments were applied within 20 min post wounding as described above. All wounds, including untreated control, were covered with a polyurethane film dressing (Tegaderm; 3M, Saint Paul, MN, United States).

### Assessment of Bacterial Wound Load

Four wounds per treatment group, per animal were biopsied with a 10 mm punch biopsy on days 2 and 5 post treatment application (*n* = 8 per assessment time), as well prior to treatment 24 h post-infection for baseline assessment. Each punch biopsy was taken around the entire wound and deep enough to remove subcutaneous tissue in order to evaluate bacteria on the wound edges, bed and surface. The biopsy was weighed and immediately placed in 1 mL of cold phosphate buffer saline (PBS) and homogenized in a sterile homogenization tube (Tenbroeck Tissue Grinder). The sample was then combined with an additional 4 mL of cold PBS. Serial dilutions were made and solutions were quantified using the Autoplate 4000 Spiral Plater System (Spiral Biotech, MA, United States). Oxacillin Resistance Screening Agar (ORSAB; Remel Inc., CA, United States) was used to quantify MRSA USA 300 and *Pseudomonas* Agar-base with CN supplementation (Remel Inc., CA, United States) was used to quantify *P. aeruginosa* 09-010 from the wounds. Plates were incubated aerobically for 24–48 h at 37°C, the colonies were counted and the log CFU/g calculated. The data was analyzed for significance using a one-way ANOVA (IBM SPSS Statistics 22) for the mean log CFU/g.

### Histological Assessment

Eight (8) biopsies from each treatment group were taken on days 5, 7, and 10 post-treatment. Biopsies were taken as an excisional biopsy (passing through the center of wounds, including normal tissue on each end). Excisional biopsies were placed in formalin then stained with hematoxylin and eosin (H&E). The samples were then analyzed and evaluated for the following parameters to determine the effects of cyclic lipopeptides on wound healing: percent of wound epithelialized (length of wound surface that was covered with epithelium, expressed as a percentage of the total length); epithelial thickness (cell layers μm); thickness of epithelium measured from five equal-distance points from each other in the biopsy and averaged; white cell infiltrate measured by the presence and amount of subepithelial mixed leukocyte infiltrates (mean score: 1 = absent, 2 = mild, 3 = moderate, 4 = marked, 5 = exuberant); granulation tissue formation; the approximate amount of new granulation tissue that formed was graded as follows: 0 = 0%, 0.5 = 1–10%, 1 = 11–30%, 2 = 31–50%, 3 = 51–70%, 4 = 71–90%, 5 = 91–100%.

### Gene Expression Analysis

Biopsies (4 mm) were also taken from each wound on days 5, 7, and 10 post-treatment for gene expression analyses associated with the wound healing process and host inflammatory response. RNA was isolated using Direct-zol RNA Mini Kit (Zymo Research, CA, United States) per company protocol. Real-Time PCR (qPCR) was performed using a One-Step RT-PCR Kit (Quanta) and run on a CFX96 Real-Time PCR System (Bio-Rad, CA, United States) to detect expression levels of the following genes: interleukin-1α (IL-1α), interleukin-8 (IL-8) and tumor necrosis factor α (TNF-α). Relative expression was normalized to housekeeping gene GAPDH. Primer sequences used were: GAPDH, forward GAPDH, forward (5′-ACATCATCCCTGCTTCTAC-3′) and reverse (5′-TTGCTTCACCACCTTCTTG-3′); IL-1α, forward (5′-GCCAATGACACAGAAGAAG-3′) and reverse (5′-TCCAGGTTATTTAGCACAGC-3′); IL-8, forward (5′-GAC CAGAGCCAGGAAGAGAC-3′) and reverse (5′-GGTGG AAAGGTGTGGAATGC-3′); TNF-α forward (5′-CACGCTCTT CTGCCTACTG-3′) and reverse (5′-ACGATGATCTGAGT CCTTGG -3′).

### Statistical Analyses

Microbiology data was analyzed for significance using a one-way ANOVA (IBM SPSS Statistics 22) for the mean log CFU/g. Gene expression data for each treatment group was analyzed using GraphPad and statistical analysis was performed using *T*-test (IBM SPSS Statistics 26). Error bars in all figures are reported as a SEM of the mean. Differences were regarded as statistically significant for *p* values of less than 0.05.

## Results

### Peptide Synthesis

Large scale tea bag synthesis of 2605-4 and 2612-8.1 was carried out to provide for the porcine model studies. The tea bag (13cm × 11cm) and one Gram of TentaGel XV RAM Fmoc (Rapp Polymere) with a substitution level of 0.24 mmol/1000 mg was used for synthesis. The mass of the crude peptide obtained post synthesis was percent yield of crude CLP 2605-4 (Mol wt: 1079.34) was 407.3 mg, resulting in crude percent yield of 78.6%, which was reduced to approximately 32% post RP-HPLC (Supplementary Data). As for the CLP 2612-8.1 (Mol wt:1281.55) was 451.2 mg, resulting in the crude percent yield of 73.3%, which was reduced to approximately 30% post RP-HPLC. The molecular weights of the CLPs was confirmed using Shimadzu ESI LC-MS and Bruker MALDI-TOF (Supplementary Data).

### Antibacterial Activity of CLPs *in vitro*

Data was obtained post 18 h incubation of the CLP’s with either Gram-positive or Gram-negative bacteria. The data suggests that the CLP 2605-4 with hydrophobic unnatural amino acid (homophenylalanine) in the macrocycle was the most effective against Gram-positive *S. aureus* Mu50 (MRSA) ATCC 700699, MIC 90: 3.1–6.25 μg/mL. It was also effective against *A. baumannii* ATCC 19606 with an MIC 90 of 6.25–12.5 μg/mL. Whereas the CLP 2612-8.1 with the two cationic exocyclic residues (diaminobutyric acid), proved to be most efficacious against *P. aeruginosa* ATCC-27853 (MIC 90: 12.5–25 μg/mL) and had comparable efficacy to that of 2605-4 against *A. baumannii* ATCC 19606, MIC 90 of 6.25–12.5 μg/mL (Table 1). The addition of hydrophobic unnatural amino acids to the macrocycle lead to increased efficacy against Gram-positive bacteria, whereas addition of positively charged exocyclic residue such as diaminobutyric acid lead to similar or increased activity against Gram-negative bacterial strains. We hypothesize that addition of above exocyclic residues added additional positive charge and increased flexibility between the macrocycle and the tail. This allowed the molecule to freely interact with the bacterial membranes, thus resulting in non-specific broad-spectrum activity.

**TABLE 1:** Antibacterial activities of all the lead new cyclic lipopeptides 2605-4 and 2612-8.1 are reported in comparison with colistin and vancomycin in μg/mL.

**Cyclic lipo-peptides (CLP)**	**MIC 90 (μg/mL)**		
	**MRSA**	***P. aeruginosa***	***A. baumannii***
2605-4	3.1–6.25	>100	6.25–12.5
2612-8.1	12.5–25	12.5–25	6.25–12.5
Colistin	>100	*ND	<0.78
Vancomycin	12.5-25	*ND	>100

*ND: Not determined.

### Peptide 2605-4 Exhibited Antimicrobial Activity Against MRSA *in vivo*

**FIGURE 2:**
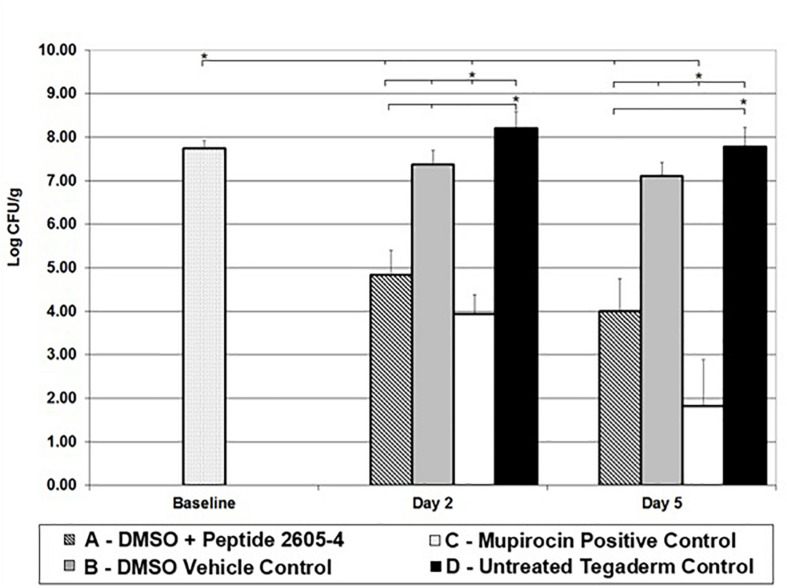
CLP 2605-4 treatment results in significant reduction of MRSA USA300 in the porcine wounds. Reduction of MRSA CFU observed in different treatment groups on various days (5, 7, and 10); *p < 0.05 (*n* = 8 per each treatment group, per time point).

Baseline wounds retrieved 24 h after infection, prior to treatment had MRSA counts of 7.75 ± 0.18 log CFU/ml. By day 2, wounds left untreated reached higher bacterial counts than the baseline samples (Figure 2). Wounds treated with only DMSO vehicle control exhibited counts similar to the baseline samples, significantly lower (*p* < 0.05) than untreated control wounds. Also, on day 2, wounds treated Peptide 2605-4 (20 mg/ml) showed a bacterial count significantly lower than all other treatment groups (*p* < 0.05), except for wounds treated with Mupirocin. Wounds treated with peptide 2605-4 showed bacterial reductions of at least 99.88% when compared to baseline CFU levels and untreated control wounds. Wounds with the smallest amount of MRSA present on day 2 were those treated with Mupirocin, with bacterial reductions of at least 99.98% compared to baseline and untreated control wounds. The largest difference between bacterial counts was observed between mupirocin and untreated control wounds.

### Peptide 2612-8.1 Exhibited Antimicrobial Activity Against *P. aeruginosa in vivo*

**FIGURE 3:**
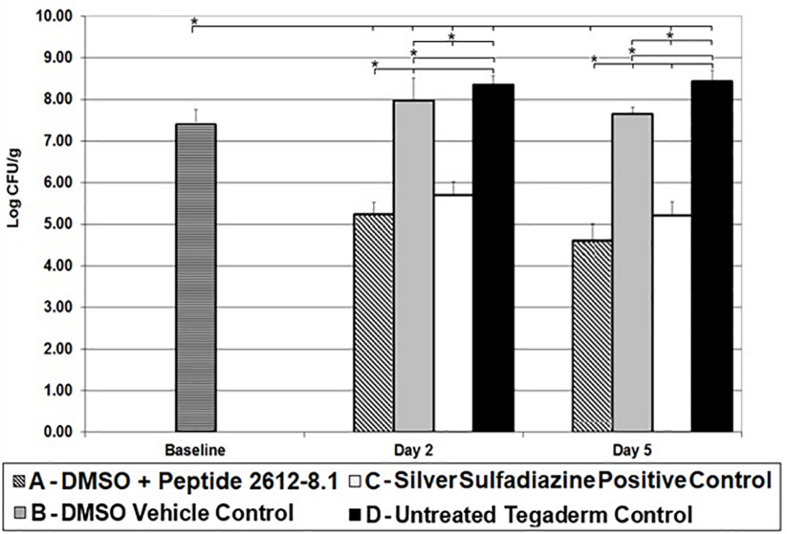
CLP 2612-8.1 treatment results in significant reduction of *P. aeruginosa* in the porcine wounds. Reduction of *P. aeruginosa* CFU observed in different treatment groups on various days (5, 7, and 10); *p < 0.05 (*n* = 8 per each treatment group, per time point).

Baseline wounds on animals infected with *P. aeruginosa* had bacterial counts of 7.40 ± 0.36 log CFU/g after 24 h. By day 2, wounds treated with only DMSO and untreated control wounds had higher bacterial counts than baseline wounds. Untreated wounds had *P. aeruginosa* load significantly higher than baseline wounds. A similar trend was seen in wounds treated vehicle (DMSO) only, with a bacterial load significantly higher than baseline wounds (Figure 3). Both, peptide 2612-8.1 and positive control silver sulfadiazine resulted in a significant bacterial load reduction compared to all other treatment groups, but no significant difference was observed between these two groups. Wounds treated with silver sulfadiazine showed counts yielding bacterial reductions of 97.99 and 99.78%, respectively, when compared to baseline and untreated wounds. Wounds treated with peptide 2612-8.1 had the lowest bacterial counts on day 2 (Figure 3), resulting in bacterial reductions of 99.30% when compared to baseline and untreated control wounds.

### Treatment With Peptide 2605-4 Resulted in Inhibition of Wound Healing, While Peptide 2612-8.1 Did Not Impede Healing Process

**FIGURE 4:**
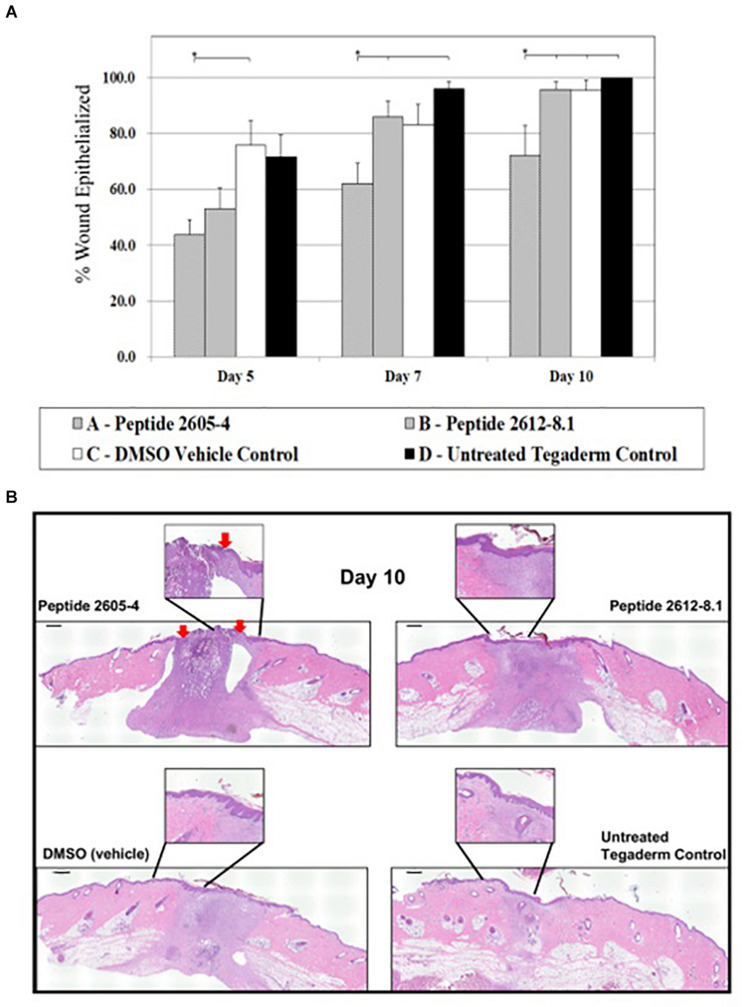
CLP 2605-4 treatment inhibited wound closure. **(A)** Graph showing percentage of epithelialization for all treatment groups at days 5, 7, and 10 post-treatment. The amount of re-epithelialized tissue was calculated as a percentage of the wounded area that was covered by newly formed epidermis. Treatment with CLP 2605-4 resulted in inhibition of epithelialization compared to other treatment groups (*p ≤ 0.05). **(B)** Representative wounds at day 10 stained with H&E are shown. Red arrows indicate extent of wound edges in CLP 2605-4 treated wounds indicating wound gap. Enlargements show epidermis fully covering wounds treated with CLP 2612-8.1, vehicle and untreated control wounds. Scale bar = 1000 μm.

To assess full therapeutic potential of the topical application of CLPs we evaluated effects on the wound healing process in the absence of infection. The amount of re-epithelialized tissue was calculated as a percentage of the wounded area that was covered by newly formed epidermis and had one or more layers of keratinocytes. This indicates the speed of newly migrating keratinocytes, which is one of the first steps to re-epithelialization of wounded tissue and represent primary outcome of wound healing in both humans and pigs. On day 5 post-treatment, wounds treated with DMSO (vehicle) displayed a significantly higher percentage of re-epithelialized tissue (75.7%) compared to wounds that were treated with CLP 2605-4. By day 7 post-treatment, untreated wounds had the highest percentages of re-epithelialized tissue at 96.0% and CLP 2612-8.1 reached 85.9%. Wounds that were untreated and those treated with peptide 2612-8.1 showed significantly more (*p* < 0.05) re-epithelialization than wounds treated with peptide 2605-4. As of day 10, all wounds had exceeded 90% re-epithelialization except for those treated with CLP 2605-4, which only reached 72.2% re-epithelialization and were significantly lower than all other treatment groups (Figure 4). Wounds treated with peptide 2612-8.1 had higher percentages of re-epithelialization compared to wounds treated with peptide 2605-4 and was able to achieve over 90% re-epithelialization by day 10, showing it has more potential as an effective antimicrobial to promote wound healing. Wounds treated with peptide 2612-8.1 showed similar percentages of re-epithelialization compared to wounds treated with DMSO alone, or those that were left untreated and only covered with a polyurethane film dressing. Modifying the structure of peptide 2612-8.1 could potentially create a compound that could allow for faster migration of new keratinocytes to the wound area, thereby shortening time to full wound closure.

**FIGURE 5:**
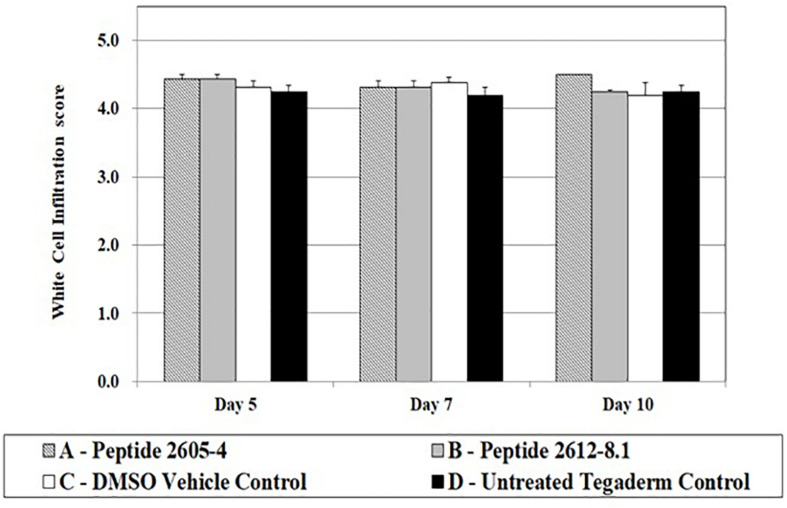
White cell infiltrate quantification upon treatment. White cell infiltrate was measured by the presence and amount of subepithelial mixed leukocytic infiltrates (mean score: 1 = absent, 2 = mild, 3 = moderate, 4 = marked, 5 = exuberant) at days 5, 7, and 10 post-treatment. No significant differences were seen among treatment groups; *n* = 8 per each treatment group, per time point.

White cell infiltration (WCI) is used to identify the inflammatory responses that could be caused by the normal processes of wound repair, microbial infection, or by the tissue reacting to the presence of foreign materials in the wound. This is also an indicator of how wound healing may progress, as excessive inflammation is detrimental to the wound healing process. All treatment groups showed WCI scores ranging from 4.2 to 4.5 (Figure 5). Wounds treated with peptide 2605-4 showed the highest score (4.5) for the entire study, however, no statistical differences were noted among treatment groups.

**FIGURE 6:**
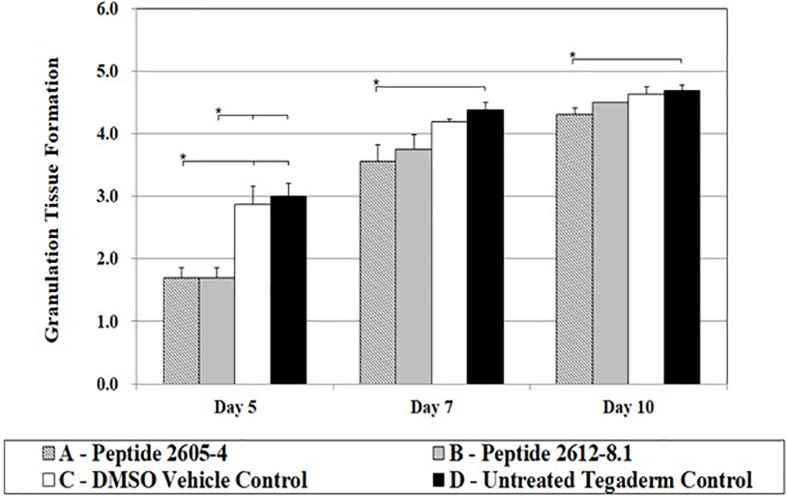
CLP treatment affected granulation tissue formation. Comparative scores of granulating wound bed assessed on days 5, 7, and 10 from tissue section stained with H&E (1 = ≤ 5%, 2 = 6–25%, 3 = 26–50%, 4 = 51–75%, 5 = 76–100%). Statistically significant difference in granulation tissue formation was observed during progression of wound healing. Reduced levels of granulation tissue were observed on day 5 in CLP 2605-4 and CLP 2612-8.1 treated wounds compared to vehicle and untreated control (*p < 0.05). Statistically significant reduction in granulation tissue formation was also found in CLP 2605-4 treated wounds compared to vehicle and untreated control at days 7 and 10 (*p < 0.05). *n* = 8 per each treatment group, per time point.

About 3 to 4 days after tissue injury granulation tissue formation begins, the hallmark process of dermal reconstitution. During this process, new blood vessels (angiogenesis), accumulation of fibroblasts, and extracellular matrices of collagen begin to form. The entire process is measured by the percent of a wound bed that has filled with newly formed granulation tissue. On day 5, peptide treated wounds were significantly lower compared to wounds treated with DMSO (vehicle) or untreated wounds, by nearly half the score (Figure 5). However, by day 7, all wounds had reached a granulation tissue formation score higher than 3, and untreated wounds showed a significantly higher (*p* < 0.05) score than those treated with Peptide 2605-4 (Figure 6). On day 10, all wounds had surpassed a granulation tissue formation score of 4, with untreated wounds continuing to show the highest score and being significantly (*p* < 0.05) higher than wounds treated with peptide 2605-4. Both peptide 2605-4 and peptide 2615-8.1 showed delayed onset and development of granulation tissue, compared to control wounds.

### Peptide 2605-4 Modulated Cutaneous Immune Response

**FIGURE 7:**
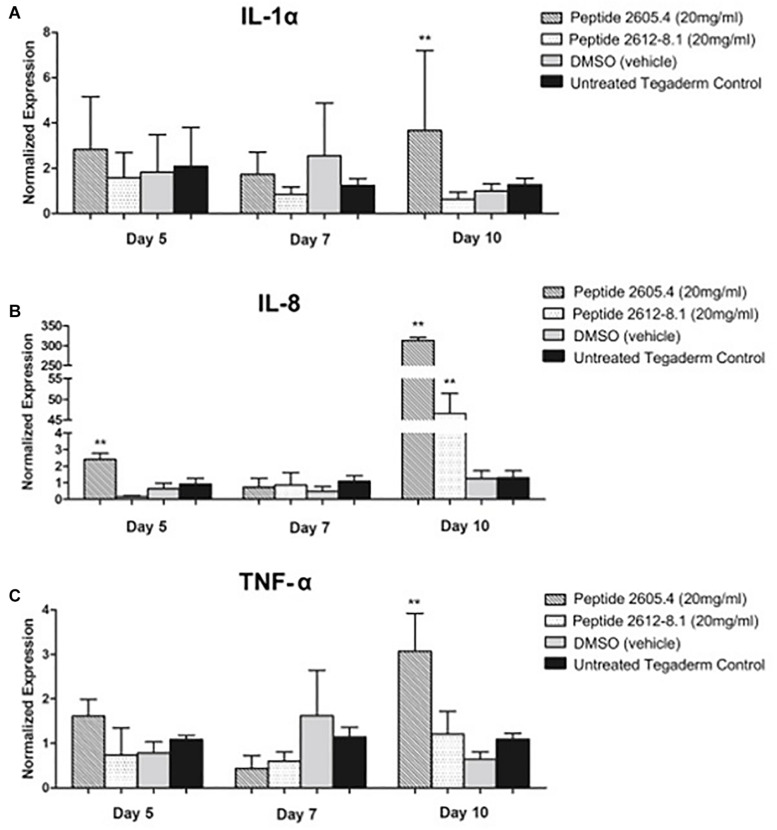
Expression of pro-inflammatory cytokines IL-1α, IL-8 and TNF-α was induced and prolonged in wounds treated with CLP 2605-4. Expression of IL-1α **(A)**, IL-8 **(B)** and TNF-α **(C)** was assessed by qPCR on days 5, 7, and 10 post-treatment. The remaining text will be keep the same (see attached pdf and word version). Mean values of were presented after normalization to GAPDH (*n* = 8 wounds per each treatment group). Error bars indicate SEM. Statistically significant differences were defined compared to vehicle control treated wounds; **p < 0.01.

Due to effects of CLP 2605-4 and 2615-8.1 on epithelialization and granulation tissue formation observed by histology, we proceeded to analyze host inflammatory response during the process of wound healing. Gene expression levels of major cytokines, IL-1α, IL-8, and TNF-α involved in inflammatory phase of wound healing were analyzed on days 5, 7, and 10 post-treatment corresponding to inflammatory, proliferative and remodeling phase of wound healing process, respectively (Vukelic et al., 2011; Eming et al., 2014; Pastar et al., 2018). IL-1α is an important pro-inflammatory cytokine released during the onset of an inflammatory response. On days 5 and 7 post-treatment, comparable expression levels of IL-1α were shown by wounds from all treatment groups, corresponding to the inflammatory phase of wound healing (Figure 7A). However, on day 10, which is the time point corresponding to remodeling phase of wound healing when inflammation should be ceased, wounds treated with peptide 2605-4 were still showing very high levels of IL-1α expression compared to peptide 2612-8.1, vehicle and untreated controls, all of which had markedly reduced levels of IL-1α expression. This data suggests wounds treated with peptide 2605-4 were not transitioning from the inflammatory phase to the proliferative and remodeling phase of the wound healing process, contributing to the delayed wound closure seen in histopathology analyses. Contrary to this, at day 10 wounds treated with peptide 2612-8.1 showed suppression of IL-1α expression, comparable to levels seen in wounds treated with DMSO and untreated wounds. Similar pattern of was observed for the IL-8 and TNF-α expression. IL-8 is a chemoattractant produced by neutrophils during the early inflammatory phase of the wound healing process.

At day 5, the highest expression levels of IL-8 were seen in wounds treated with peptide 2605-4 (Figure 7B). Overall, the expression of IL-8 was lower by day 7, however, by day 10 there was a large induction of IL-8 expression in wounds that were treated with either peptide 2605-4 or peptide 2612-8.1. Of these two, peptide 2605-4 had the highest level of IL-8 expression, correlating to the delayed healing and prolonged inflammatory observed in wounds treated with peptide 2605-4, along with the lower scores for granulation tissue formation seen in wounds treated with either peptide 2605-4 or peptide 2612-8.1.

On day 5, wounds treated with peptide 2605-4 had also significantly higher (*p* < 0.01) levels of TNF-α expression, compared to all other treatment groups, while wounds treated with peptide 2612-8.1 showed the lowest level of this pro-inflammatory cytokine mainly produced by neutrophils and macrophages (Figure 7C). On day 7, wounds from all treatment groups had comparable levels of TNF-α expression and wounds treated with peptide 2605-4 showed reduced levels of TNF-α. However, by day 10 wounds treated with peptide 2605-4 displayed a significant (*p* < 0.01) increase in TNF-α expression, whereas peptide 2612-8.1, vehicle, and untreated wounds were comparable to previous time points. Wounds treated with peptide 2605-4 had the highest expression of TNF-α, correlating to the induction of both IL-1α and IL-8.

Altogether, high expression levels of these pro-inflammatory cytokines in wounds treated with peptide 2605-4 at day 10 of the study supports the prolonged, unresolved inflammation and inhibition of wound healing, as confirmed by the histopathology results above. The histopathology results and representative H&E staining at day 10 further corroborate gene expression analyses and prolonged inflammatory response seen in wounds treated with peptide 2605-4. More pronounced hematoxylin (purple) staining in wounds treated with peptide 2605-4 suggests more severe inflammation, which corresponds to the expression levels of pro-inflammatory cytokines (Figure 7). Untreated control wounds showed almost complete remodeling as expected while vehicle (DMSO) treated wounds showed tissue remodeling, but to a lesser extent than untreated control wounds.

## Discussion

The alarming spread of multi drug-resistant *S. aureu*s and *P. aeruginosa* causing wound infections, has broadened the search for viable antimicrobials that will prove beneficial to wound healing while also controlling infections (Banin et al., 2017). A novel class of compounds, CLPs, have come to light as a new class of antimicrobial compounds. These drugs are able to disrupt and pass through the bacterial membrane, but in a manner different from contemporary cationic antimicrobials (Cochrane and Vederas, 2016). Our study focused on the synthesis of novel CLP 2605-4 and 2612-8.1 and evaluation of their ability to heal wounds while controlling MRSA and *P. aeruginosa* infections *in vivo*. Peptide 2612-8.1 was effective at controlling *P. aeruginosa* infections and was able to promote wound healing that was analogous to DMSO-treated and untreated control wounds. Peptide 2605-4 was able to reduce bacterial counts of MRSA *in vivo* but was not effective at healing full-thickness wounds, as evidenced by the over-expression of pro-inflammatory cytokines, and histopathology results showing prolonged inflammation, lower rates of re-epithelialization and reduced granulation tissue formation. While few cyclic lipopeptides have met FDA approval, those that have met approval show promising effects, leading to further investigations of CLPs as a new class of antimicrobial compounds, particularly against drug-resistant bacteria associated with hospital acquired infections (Murray et al., 2006).

Daptomycin is secondary metabolic produced by *Streptomyces roseosporus* during fermentation and is used to treat complicated skin and skin structure infections (cSSSIs) of *S. aureus* (Richards and Melander, 2009). It is highly bactericidal, killing 99.9% of bacteria within 1 h after treatment (Miao et al., 2005; Meena and Kanwar, 2015). The mechanism of action daptomycin uses is unique to conventional antimicrobial compounds, acting in a calcium-dependent manner that allows daptomycin to oligomerize in the bacterial cell wall, creating pores that cause destabilization of the electrical membrane potential (Fuchs et al., 2002; Silverman et al., 2003; Cha and Rybak, 2004). This leads to inhibition of peptidoglycan synthesis, and ultimately halts transcription of DNA. Pre-clinical trials showed effective antimicrobial activity against numerous strains of drug-resistant and -sensitive Gram-positive bacteria, and was able to quell infections in various tissues of experimental animals, showing a wide range of usage (Kaatz et al., 1990; Alder et al., 2003; Cha et al., 2003; Dandekar et al., 2003; Steenbergen et al., 2005; Straus and Hancock, 2006). Clinical studies revealed daptomycin to be comparable to standard therapy, while being non-inferior, leading to FDA approval in the United States in 2003. These clinical studies developed a safe and effective dosage of 4 mg/kg that is administered once daily, and is marketed in the United States as Cubicin^®^ by Cubist Pharmaceuticals (Louie et al., 2001; Bionda and Cudic, 2011). To date, daptomycin is the only FDA-approved cyclic lipopeptide antimicrobial compound.

Despite the lack of government-approved CLPs, the efficiency of compounds such as daptomycin, as well as the broad spectrum of these secondary metabolites, has highlighted the potential for discovering new CLPs that have antimicrobial activity. Topical application of CLPs for the treatment of wound infections has only recently been considered (Bionda et al., 2014). Regardless of wound type, controlling bacterial infection is important and can affect the progression and time it takes for wounds to heal, especially if the bacteria is capable of forming a biofilm in the wound (Nishikioro et al., 1986). Using the *in vitro* approach, we determined that CLP 2605-4 is more effective against MRSA USA-300 while CLP 2612-8.1 has shown to be effective against *P. aeruginosa* (Table 1).

Our *in vivo* studies confirmed efficacy of CLP 2605-4 against, MRSA drug-resistant bacteria frequently recovered from combat wounds (Hussein, 2019). Wounds treated with CLP 2605-4 showed significant reductions in bacterial counts 48 h post-treatment through day 5, compared to vehicle and untreated control wounds. However, the positive control Mupirocin formulated for topical application was the most successful at reducing MRSA, suggesting that development of topical vehicle for CLP 2605-4 may enhance observed antimicrobial activity. CLP 2612-8.1 has shown to be effective against *P. aeruginosa in vivo* decreasing the bacterial count in at least 3 logs compared to untreated control wounds. While the CLP 2605-4 was effective at reducing MRSA counts in full-thickness wounds, it lagged in its ability to aid the wound healing process compared to other treatments, as discussed below.

Inflammation is a critical process that takes place shortly after wounding, and effects the progression of wound healing (Strbo et al., 2014; Landén et al., 2016). Gene expression analysis of several pro-inflammatory cytokines was performed to determine inflammatory responses to wound treatments. After treatment, IL-1α expression reduced to levels comparable among all treatment groups, except in wounds treated with Peptide 2605-4, where it remained elevated. Wounds treated with CLP 2612-8.1 showed the lowest levels of IL-1α at days 7 and 10. It has been shown that inhibiting the expression of IL-1α leads to improved wound healing (Ishida et al., 2006; Thomay et al., 2009), while our data indicate potential anti-inflammatory effect of CLP 2612-8.1, warranting further investigation. At day 10, however, IL-1α levels increased significantly in wounds treated with CLP 2605-4 compared to all treatment groups. After cutaneous injury tissue, pre-stored IL-1α released by keratinocytes to promote neutrophil recruitment from circulation to the injured tissue (Pastar et al., 2014). Once having entered the injured tissue, neutrophils become the predominant source of IL-1α expression, where it aids in bacterial clearance. However, expression of IL-1α is tightly controlled during wound healing and suppressed after inflammatory phase is completed to allow progression to proliferative phase of the tissue repair and complete wound closure. Increased expression of Il-1α in the wounds treated with CLP 2605-4 was contributing to impaired re-epithelialization and reduction of granulation tissue formation (Figure 6). IL-8 expression was also significantly higher in wounds treated with peptide 2605-4. Although IL-8 expression was reduced by day 7, at day 10, expression levels in wounds treated with CLP 2605-4 or CLP 2612-8.1 rose significantly compared to control treatments. IL-8 is a chemoattractant produced by neutrophils during the early inflammatory phase of the wound healing process. IL-8 expression is also highest during the initial inflammatory responses of wound healing and suppression of IL-8 is required for advancing into the proliferative phase of wound healing. Therefore, the prolonged induction of IL-8 caused by CLP 2605-4, together with increased expression of IL-1α may contribute to observed deficiency in wound re-epithelialization and granulation tissue formation. At the beginning of the study, tumor necrosis factor-α (TNF-α) levels were highest in wounds treated with Peptide 2605-4, but not significantly higher than the other treatment groups. By day 10, wounds treated with CLP 2605-4 had demonstrated significant increases in TNF-α expression, compared to CLP 2612-8.1 and control. TNF-α is a pro-inflammatory cytokine produced mainly by neutrophils and macrophages during the initial inflammatory response during wound healing. TNF-α is both beneficial and detrimental to the wound healing process, depending on the dose, onset, and duration of TNF-α exposure to wounded tissue (Barrientos et al., 2008).

Inability to control the inflammatory response delays wound healing by preventing proper tissue repair (Behm et al., 2012; MacLeod and Mansbridge, 2016). Elevated expression of pro-inflammatory cytokines in wounds treated with CLP 2605-4 coordinate with histopathology results demonstrating lower percentages of re-epithelialized tissue and less granulation tissue formation. Despite being able to reduce MRSA USA-300 wound load *in vivo*, CLP 2605-4 does not appear to be an adequate treatment for wound healing. Whether this is due to the presence of bacterial virulence factors, or perhaps an effect of the peptide on immune cells within the wound environment, is unknown. Effects of peptides on cytokine expression have been showed in previous studies suggesting that peptides contribute with the immunomodulatory and anti-inflammatory activities (van Dijk et al., 2016; Bonvini et al., 2018). Our results suggested that 2612-8.1 had better antimicrobial activity and lowered the inflammatory response, interesting similar evidence were published for Kolls et al. (2008). Further studies are needed to determine whether this is due to the increased bacterial virulence as a response to partial kill, or an effect of the peptide on the host cells within the wound environment. Interestingly number of white cell infiltrates was unaffected by CLP treatment regardless of the day of assessment, suggesting that other cell types involved in wound healing such as fibroblasts, keratinocytes or endothelial cells may be a source of IL-1α, IL-8, and TNF-α.

The need for developing novel compounds that promote wound healing while simultaneously combating infection is of ultimate importance as the repertoire of MDR bacteria increases. Our findings strengthen the need for innovative compounds that are effective as both antimicrobials and agents promoting wound healing.

## Data Availability Statement

The raw data supporting the conclusions of this article will be made available by the authors, without undue reservation.

## Ethics Statement

The animal study was reviewed and approved by the Institutional Animal Care and Use Committee (IACUC), University of Miami.

## Author Contributions

JG, IP, SP, RH, PC, and SD contributed to conception and design of the study. JG, AH, MS, and CH organized the database. JG, IP, and CH performed the statistical analysis. JG, AH, MS, JV, and CS perform the study with animals. IP and CH perform the molecular analysis. SP, RH, and PC work in the development of different formulations. JG, IP, PC, and SD wrote the sections of the manuscript. All authors contributed to manuscript revision, read, and approved the submitted version.

## Conflict of Interest

The authors declare that the research was conducted in the absence of any commercial or financial relationships that could be construed as a potential conflict of interest.

## Publisher’s Note

All claims expressed in this article are solely those of the authors and do not necessarily represent those of their affiliated organizations, or those of the publisher, the editors and the reviewers. Any product that may be evaluated in this article, or claim that may be made by its manufacturer, is not guaranteed or endorsed by the publisher.
