# Supplementary material for: Novel Cyclic Lipopeptides Fusaricidin Analogs for Treating Wound Infections
Source: Front Microbiol. 2021 Jul 23;12:708904. doi: 10.3389/fmicb.2021.708904 (PMC8343139; doi:10.3389/fmicb.2021.708904)
Supplement: Supplementary file 1 [file Data_Sheet_1.pdf]

Supplementary Data

Purification of Peptides:

During purification, the peak molecular weight corresponding to the desired product molecular weight, calculated using ChemBioDraw Ultra 12.0 software, was isolated. The substances were purified using by RP-HPLC in Agilent Technologies 1260 infinity preparative HPLC system. The system features included a G1361A 1260 prep binary solvent pumps, a G1364B 1260 FC-PS fraction collector, and a G1365D 1260 MWD VL detector with its internal wavelength set to both 214 nm and 254 nm. The separations were carried out on a Luna Omega C18 preparative column (5 µm, 250 mm × 21.5 mm i.d.) with an anteriorly connected Luna Omega C18 column guard (5 µm, 15 mm × 21.5 mm i.d.). The mobile phase solvents included 0.1% formic acid solutions of water (solvent A) and acetonitrile (solvent B). The mobile phases for HPLC purification were HPLC grade obtained from Sigma Aldrich and Fisher Scientific. The samples were dissolved in 30% acetonitrile, sterile filtered and subjected to a linear increase in gradient 25-65% (v/v) of solvent B over a 40 min method for enhanced separation. The total flow rate was set to 10mL/min and the total injection volume was set to 3000uL.The retention times were approximately 17.8 and 21 mins for 2605-4 and 2612 8.1 respectively.

LCMS analysis

Purity and identity of the peptides were also verified using a Shimadzu 2010 LCMS system, Shimadzu LCMS solutions software version 3, consisting of a LC-20AD binary solvent pumps, a DGU-20A degasser unit, a CTO-20A column oven, and a SIL-20A HT auto sampler. A Shimadzu SPD-M20A diode array detector was used for detections. The spectral wavelengths were set to 214nm and 254nm. The Chromatographic separations were obtained using a Phenomenex Luna C18 analytical column (5µm, 50 x 4.6mm i.d.). The column was protected by a Phenomenex C18 column guard (5µm, 4 x 3.0mm i.d.). The mobile phases consisted of a mixture of LCMS grade Acetonitrile /water (both with 0.1% formic acid). Mobile phases used were either HPLC grade or LCMS grade obtained from Sigma Aldrich and Fisher Scientific. The initial setting for analysis was 5% Acetonitrile (v/v), then linearly increased to 55% Acetonitrile over 12 minutes. The gradient was then held at 95% Acetonitrile for 2 minutes before being linearly decreasing to 5%.

The flow rate was adjusted to 0.5mL/minute. The column oven and flow cell temperature for the diode array detector was 30°C. The auto sampler temperature was held at 15°C. and Sample dilutions were made in 50% (v/v) of acetonitrile:water and 5uL of sample was injected for analysis.

Peptides (CLP's)	Molecular formula	Molecular weight(actual)	Molecular weight found (MALDI-TOF)	Molecular weight found (LCMS)
2605-4	$C_{56}H_{82}N_{14}O_8$	1079.34	1080.28 [M+H]+	540 [M+2/2]+
2612-8.1	$C_{62}H_{96}N_{20}O_{10}$	1281.55	1282.51 [M+H]+	641 [M+2/2]+

MALDI-TOF

The peptides (CLP's) were first dissolved in 70% acetonitrile and 30% H_2O. Then, 0.5µL of the matrix that consisted of 1 mg of α-Cyano-4-hydroxycinnamic acid (α-CHCA) in 50µL acetonitrile, 30µL methanol, and 20µL H_2O was added to the Maldi-TOF plate (MSP 96 target ground steel BC). This was followed by the addition of 0.5µL of previously diluted peptide to the matrix. The plate was scanned using Reflector Positive Ion Mode and measured a range of 700-2000 Daltons. The instrument used was Bruker Microflex LRF MALDI-TOF (Billerica, Massachusetts, USA)

2605-4

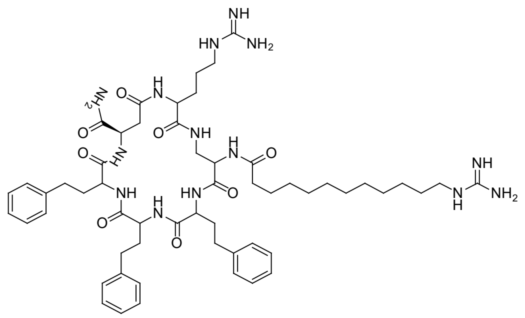

Comment 1 1% laser
Comment 2

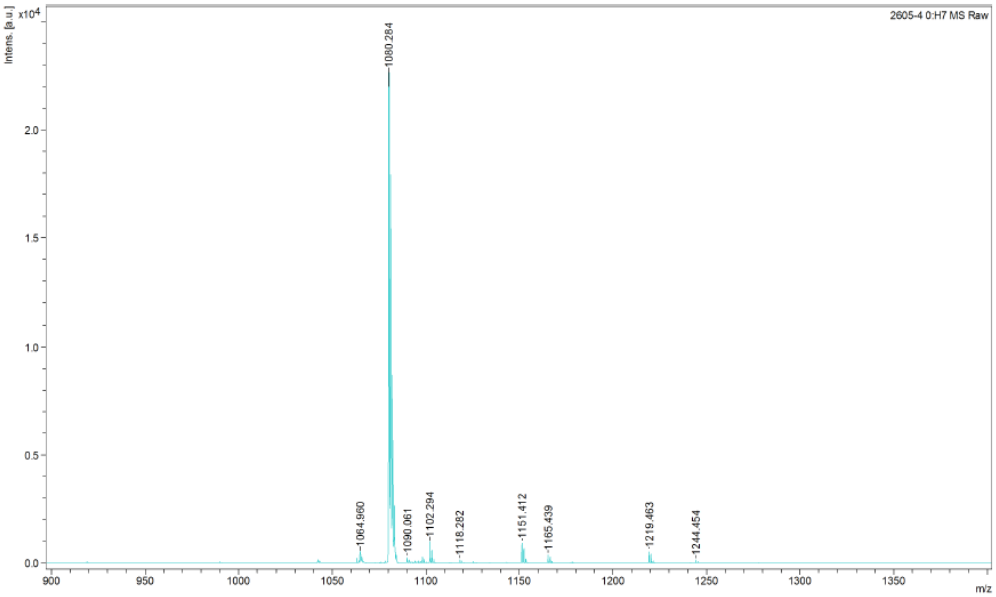

printed: 5/15/2018 3:29:02 PM Acquired: 2018-05-15T13:53:32.516-04:00 Target well: H7

2612-8.1

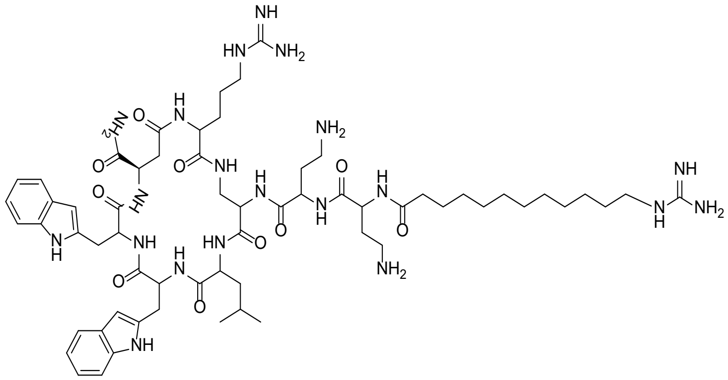

NH
HN
NH2
H
N
O
NH2
O
HN
O
NH
NH2
O
H
N
O
N
H
H
N
O
NH
NH2
N
H
NH
H
O
NH
O
H
N
O
HN
O
NH2
NH

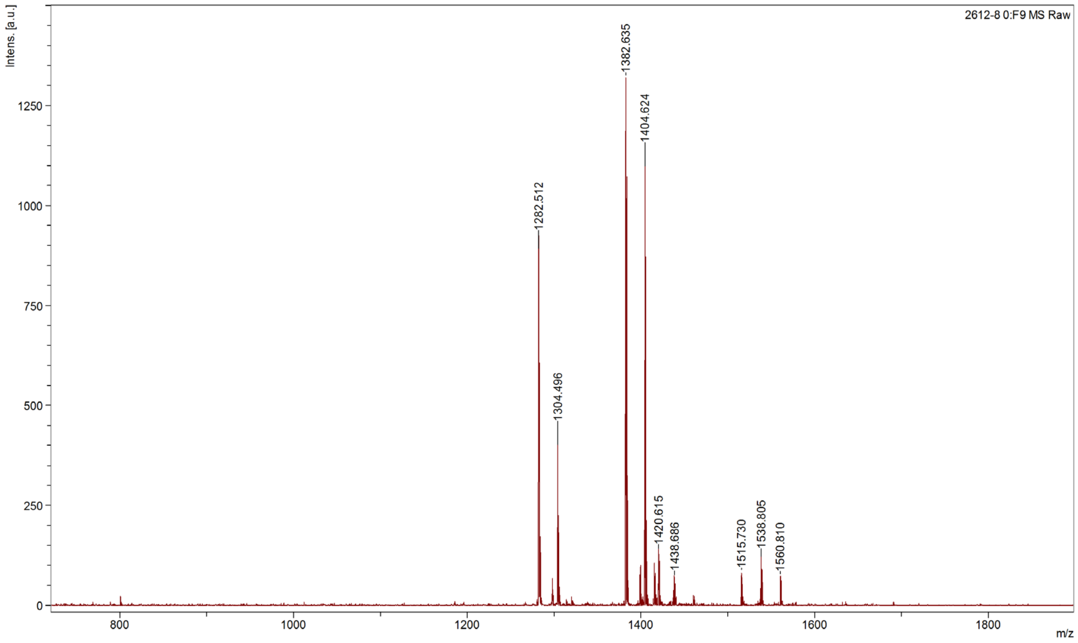

Intens. [a.u.]
2612-8 0:F9 MS Raw
1382.635
1404.624
1282.512
1304.496
1420.615
1438.686
1515.730
1538.805
1560.810
1250
1000
750
500
250
0
800
1000
1200
1400
1600
1800
m/z
